# The panacea statistical toolbox of a biomedical peer reviewer

**DOI:** 10.12669/pjms.314.8611

**Published:** 2015

**Authors:** Younis Skaik

**Keywords:** Biostatistics, Peer Reviewers

## Abstract

The main role of a peer reviewer is to make judgments on the research articles by asking a number of questions to evaluate the quality of the research article. Statistics is a major part of any biomedical research article, and most reviewers gain their experiences in manuscript reviewing by undertaking it but not through an educational process. Therefore, reviewers of the biomedical journals normally do not have enough knowledge and skills to evaluate the validity of statistical methods used in biomedical research articles submitted for consideration. Hence, inappropriate statistical analysis in medical journals can lead to misleading conclusions and incorrect results. In this paper, the most common basic statistical guidelines are described that might be a road map to the biomedical reviewers. It is not meant for statisticians or medical editors who have special interest and expertise in statistical analysis.

## INTRODUCTION

‘Statistics is the grammar of science’ by Karl Pearson, 1982.

After the passage of the research articles through the editorial quality tests, the articles are then forwarded to the gatekeepers “Peer reviewers” in a process called peer review. The reviewers are also called “authors’ peers” as they work in the same research area. The main role of a peer reviewer is to make judgments on the research articles. Each research article is evaluated by the reviewer by asking a number of questions to evaluate the quality of the research article. Major questions like what is research about?, is it interesting and important?, is the methodology sound?, are the findings original and of considerable value?, are the conclusions appropriate?. Based on the answers of those questions, the reviewers judge on the quality of the research article, and they make a recommendation to the editor, who finally decides whether to publish the research article or not. Since 1960s there has been a dramatic increase in the application of statistical methods in the biomedical sciences.^1^ Adequate designed studies, representative samples (sample size), and appropriate statistical tests are all vital to display data in a concise and representative way and to estimate the probability (P value) of making an error.^2^,^3^ Most reviewers gain their experiences in manuscript reviewing by undertaking it but not through an educational process.^4^ Therefore, reviewers of the biomedical journals normally do not have enough knowledge and skills to evaluate the validity of statistical methods used in all kinds of research articles submitted for consideration.^5^ It is then difficult to have a reviewer armed with basic and/or advanced statistical knowledge.^5^ This has been confirmed by several studies which showed that half of the published articles in the biomedical sciences have incorrect statistical methods.^6^-^9^ Hence, inappropriate statistical analysis in medical journals can lead to misleading conclusions and incorrect results. In this paper, the most common basic statistical guidelines are described that may help the biomedical reviewers.

### The panacea statistical toolbox of biomedical reviewers

The main topics of the basic statistical knowledge that may be useful for the biomedical reviewers are illustrated in Table-I.

**Table-I:** The panacea statistical toolbox of a biomedical reviewer.

• Types of data.
• Which statistical test is the most appropriate to analyze the data?
• What is the P value?
• Numerical and graphical description of data.

### 1. Types of data

The target of most studies is to collect data about a particular topic, present them in a meaningful way, and to extract information from them. Data are often discussed as variables; a variable is any characteristic that varies from one to another in a defined population. For example, weight in kilogram varies from person to person. There are two major types of variables: categorical or numerical. The importance of knowing the data type is to determine the most appropriate statistical test that can be applied to analyze the data.^1^

### Categorical (qualitative) data

Defined by the classes or categories into which an individual can belong.

### Nominal data

Name only. Examples include gender, ethnicity, blood group, or marital status.

### Ordinal data

Variable is ordered. Examples like “Yes/No” or “Low/Medium/High”.

### Numerical (quantitative) data

The variable has a numerical value.

### 1.2.1 Discrete data

a number obtained by counting. Examples include the number of biomedical reviewers who have statistical knowledge.

### 1.2.2 Continuous data

it reflects a measurement. There is no limitation on the values that the variable can take. Examples include blood pressure, height, or weight.

Transforming continuous data into two or more ordinal data is often observed in the biomedical literature to make distributions closer to normal distribution, and sometimes to make easier both interpretation of the results and data comparison.^10^ There are several methods to transform data, which all can reduce the precision of measurements and hence causes false negative or positive results.^11^,^12^ Therefore, the reviewers should pay an attention whether the authors mention how and why the transformation was done, if there is any. In addition, non-parametric tests that do not assume normal distribution are always an alternative to analyze the data rather than transforming data.

### Which statistical test is the most appropriate to analyze the data?

According to the data type, it is of utmost importance to select the most appropriate statistical test to avoid the unsound conclusions and incorrect research results. Table-II shows the most common statistical tests for simple analysis of data, which can be used by the reviewers to check the appropriateness of statistical tests used in the submitted research articles. More statistical tests which are used for regression studies, longitudinal studies, and assessing evidence are more advanced and hence are not highlighted in this paper.

**Table-II:** Most common statistical tests used for simple analysis of data.

Type of data	No. of comparing groups / Categories	Statistical test
Numerical	One group	t-test, Sign test
	Two paired (matched)	Paired t-test, Wilcoxon sign rank test, Sign test
	Two independent groups	Unpaired t-test, Wilcoxon rank sum test
	More than 2 groups	One-way ANOVA, Kruskal-Wallis test
Categorical	Two categories (1 group)	Z test
	Two categories (2 groups paired)	McNemar’s test
	Two categories (2 independent groups)	Chi-squared test, Fisher’s exact test
	Two categories (more than 2 groups)	Chi-squared test, Chi-squared trend test
	More than two categories	Chi-squared test

The research question, the data type, and the number of groups involved in the study are the main factors which play a role in selecting the most appropriate statistical test.

### What is the P value?

P value is the estimated probability but not a biological importance of occurrence of observed effect if the null hypothesis (H_0_) of a study question is true. It is used to assess the statistical significance, however, the strength of association and effect size are limitations.^13^ The reviewers should look for the exact P values in the submitted papers but not more or less the significance level (0.05 or 0.01). If the exact P-value of an observation (e.g., comparing two groups) was 0.049 with a sample size of 20, and the authors reported the P value as < 0.05, then many researchers would not think to replicate the results with a larger sample size. It is also recommended to report in addition the 95% confidence interval for the difference.^14^

### Numerical and graphical description of data

Numerical and graphical summaries of data would save the time of the readers and the data would be more informative. However, the question that should be raised by the reviewers is that which graphical methods and summary statistics would be more valuable in certain situations to avoid the incorrect usage by the authors?^15^

**Fig.1:**
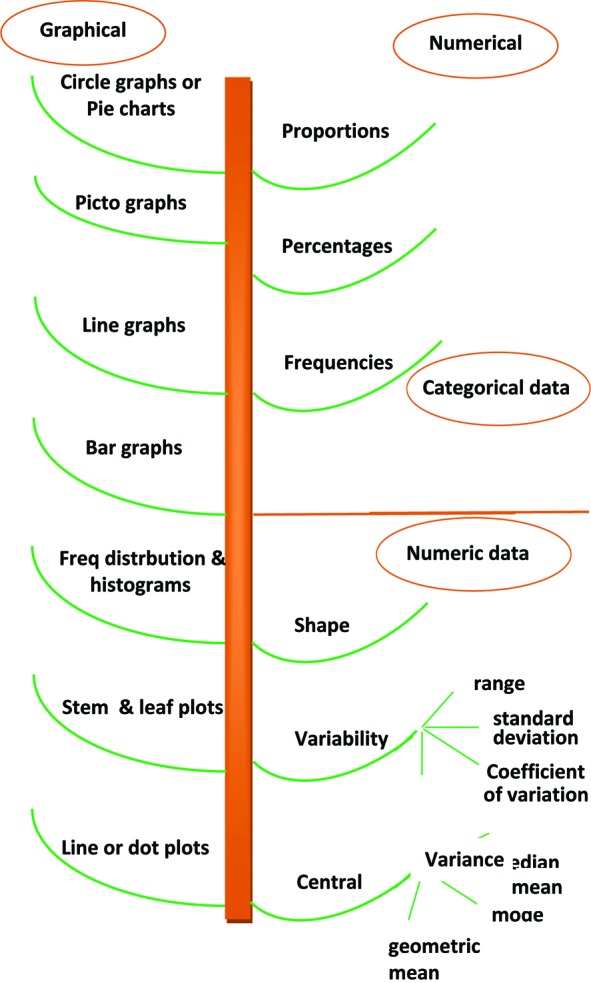
Representation of data.

Fig.1 illustrates a wide spectrum of visualizing the data, which are all important for the reviewers to have an idea about the basic concepts of each item. The reviewers should be aware of the following common mistakes in the graphical and numerical description of data and how to avoid them.

1. Most biological data are not normally distributed.^16^ Hence, mean and standard deviation (SD) are not the correct tools to describe the skewed data. Alternatively, medians and inter quartile ranges can be used in such situations.
2. The mean alone is not enough to describe the variability of data.
3. The SD and standard error of the mean (SEM) are interchangeably used in the biomedical literature despite they are different.^17^ SD is used in the summarized descriptive data to describe that quantifies variability within the sample. However, SEM is limited to compute the confidence intervals (CI) and hence quantifies uncertainty in the estimate of the mean.
4. Figures and tables to summarize the data should be presented in a way to assist the readers and not to mislead them.
5. Graphs that do not start at zero are tricky and can mislead the readers. Therefore, starting the graphs and charts at zero would give accurate comparability of the columns.
6. Scales with equals intervals are highly recommended rather than compressing or lengthened one of the axis which can lead to distorted relationship between the two axes.

## CONCLUSIONS

Notwithstanding the statistical software packages facilitate the task of data analysis for statistically unskilled researchers; major statistical problems are still determined in the biomedical research articles, due to the insufficient knowledge of researchers of the statistical ideas and mathematical concepts. Therefore, screening the submitted articles for the validity of statistical analysis of the data is an additional task of the biomedical reviewers. Thus, the reviewers should have at least basic statistical knowledge to be able to end up with sound results and correct conclusions. In addition, consulting a statistician or statistically skilled experts should be a choice for the editors and reviewers to enhance the statistical quality of the biomedical published articles.
